# Case study: osteoid osteoma - an unusual cause of shin splints

**DOI:** 10.1186/1757-1146-3-S1-P13

**Published:** 2010-12-20

**Authors:** Bob Longworth, Alex Thompson

**Affiliations:** 1 NHS, Leeds PCT, Leeds, UK

## 

A 16-year-old patient presented to a physiotherapy clinic with a classic case of ‘shin splints’. Pain had started with increasing activity as she was playing Netball for her school team. Medial Tibial Stress Syndrome (MTSS) was identified clinically and treatment started. The patient stopped her sporting activity and initial physiotherapy treatment seemed to work as the pain subsided. After 6 months the patient returned to her activities. Unfortunately, any attempt to participate in netball once again brought on her symptoms. It was noted that she had ‘flat feet’ so the Podiatrists opinion was sought. The Podiatrist suggested an X-ray to rule out a fatigue fracture. The X-ray report showed a lucent area in the mid shaft of the Left tibia, suggesting either an osteoid osteoma or infection. The patient was referred through to Orthopaedics where a further CT scan diagnosed an osteoid osteoma which was subsequently excised.

MTSS has many aetiological factors that were present in this patient (bowed tibia’s, pronated feet, high impact sport and that the patient was female). The diagnosis however was significantly different. This case highlights the need for a care pathway that involves Imaging for non-responding MTSS.

